# Feasibility and clinical utility of handheld fundus cameras for retinal imaging

**DOI:** 10.1038/s41433-021-01926-y

**Published:** 2022-01-12

**Authors:** Susmit Das, Helen J. Kuht, Ian De Silva, Sundeep S. Deol, Lina Osman, Joyce Burns, Nagini Sarvananthan, Usman Sarodia, Bharat Kapoor, Tahir Islam, Raghavan Sampath, Alicia Poyser, Vasileios Konidaris, Rossella Anzidei, Frank A. Proudlock, Mervyn G. Thomas

**Affiliations:** 1 Leicester Medical School, College of Life Sciences, University of Leicester, George Davis Centre, 15 Lancaster Rd, Leicester, LE1 7HA UK; 2 Department of Neuroscience, Psychology and Behaviour, The University of Leicester Ulverscroft Eye Unit, University of Leicester, RKCSB, Leicester, LE2 7LX UK; 3 Department of Ophthalmology, University Hospitals of Leicester, Leicester Royal Infirmary, Leicester, LE1 5WW UK

**Keywords:** Medical imaging, Medical research

## Abstract

**Background/objectives:**

Handheld fundus cameras are portable and cheaper alternatives to table-top counterparts. To date there have been no studies comparing feasibility and clinical utility of handheld fundus cameras to table-top devices. We compare the feasibility and clinical utility of four handheld fundus cameras/retinal imaging devices (Remidio NMFOP, Volk Pictor Plus, Volk iNview, oDocs visoScope) to a table-top camera (Zeiss Visucam^NM/FA^).

**Subjects/methods:**

Healthy participants (*n* = 10, mean age ± SD = 21.0 ± 0.9 years) underwent fundus photography with five devices to assess success/failure rates of image acquisition. Participants with optic disc abnormalities (*n* = 8, mean age ± SD = 26.8 ± 15.9) and macular abnormalities (*n* = 10, mean age ± SD = 71.6 ± 15.4) underwent imaging with the top three scoring fundus cameras. Images were randomised and subsequently validated by ophthalmologists masked to the diagnoses and devices used.

**Results:**

Image acquisition success rates (100%) were achieved in non-mydriatic and mydriatic settings for Zeiss, Remidio and Pictor, compared with lower success rates for iNview and oDocs. Image quality and gradeability were significantly higher for Zeiss, Remidio and Pictor (*p* < 0.0001) compared to iNview and oDocs. For cup:disc ratio estimates, similar levels of bias were seen for Zeiss (−0.09 ± SD:0.15), Remidio (−0.07 ± SD:0.14) and Pictor (−0.05 ± SD:0.16). Diagnostic sensitivities were highest for Zeiss (84.9%; 95% CI, 78.2–91.5%) followed by Pictor (78.1%; 95% CI, 66.6–89.5%) and Remidio (77.5%; 95% CI, 65.9–89.0%).

**Conclusions:**

Remidio and Pictor achieve comparable results to the Zeiss table-top camera. Both devices achieved similar scores in feasibility, image quality, image gradeability and diagnostic sensitivity. This suggests that these devices potentially offer a more cost-effective alternative in certain clinical scenarios.

## Introduction

Fundus photography is one of the most common imaging modalities used in ophthalmology for the screening, diagnosis, documentation and surveillance of retinal, optic nerve (ON) and retinal vascular abnormalities. The first sign of a systemic or life-threatening disorder could be picked up on fundus examination or photography, thus it has widespread applications beyond ophthalmology. In an ophthalmic emergency department setting, fundus photography has shown to not only augment diagnosis of optic disc oedema but also change the final diagnosis and subsequent management [1]. With the advent of tele-ophthalmology and development of artificial intelligence systems to interpret fundus photos, it is anticipated that fundus imaging will be performed outside of the ophthalmology clinics (for example emergency department, general medical clinics and general practitioners) [2–4].

Traditional table-top fundus camera systems are large and difficult to transport. Newer handheld fundus cameras utilise a smaller form factor, allowing for greater portability and ease of use [5]. These handheld cameras are available at significantly lower service costs and range from £50–4500 [6]. Moreover, due to the “point and shoot” nature of handheld devices it has the potential to be used by non-specialists [7]. To date, there have been limited comparative studies looking at feasibility and clinical utility of handheld fundus cameras in relation to traditional table-top fundus cameras [5].

In our study, we compare four handheld fundus cameras/retinal imaging device to a table-top counterpart. We assess the devices for their feasibilities of image acquisition, image quality and gradeability, and participant experience.

## Materials and methods

### Study population

Recruitment and subsequent data collection were carried out at University of Leicester Hospitals NHS trust, UK, between January 2020 – March 2020 and August 2021 – September 2021. The study was split into two stages. This included imaging of healthy participants without any ophthalmic pathology (stage 1) and participants with optic disc or macular abnormalities (stage 2). All image acquisition was performed by one examiner to ensure consistency across all tested devices. Informed consent was obtained from all participants. The study was approved by the local research and ethics committees (Leicestershire, Northamptonshire & Rutland Research Ethics Committee; REC reference: 10/H0406/74) and the research adhered to the tenets of the Declaration of Helsinki. The participant demographics are shown in Supplementary Table 1.

For stage one of the study, participants (*n* = 10, mean age ± SD = 21.0 ± 0.9 years) without any known eye conditions were recruited. Participants were excluded if there were any known ophthalmic conditions. For stage two of the study, participants with optic disc abnormalities (*n* = 8, mean age ± SD = 26.8 ± 15.9 years) and macular abnormalities (*n* = 10, mean age ± SD = 71.6 ± 15.4 years) were recruited to participate from Eye Casualty, Emergency department and outpatient clinics at the Leicester Royal Infirmary, UK. Inclusion criteria for recruitment in stage 2 included participants presenting to the eye casualty, emergency department or outpatient clinics with suspected optic disc swelling or macular abnormalities detected on fundoscopy.

### Imaging modalities

**Fig. 1:**
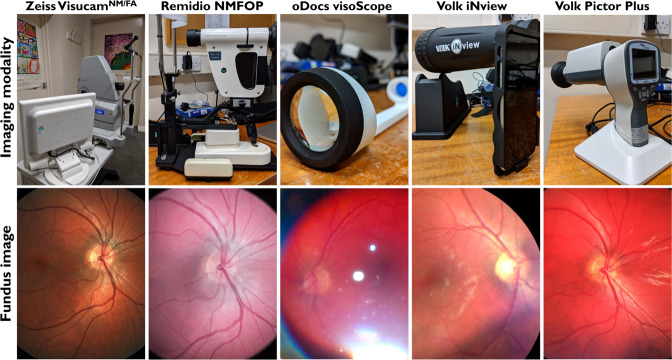
Overview of table-mounted and handheld fundus cameras/retinal imaging devices and fundus images. The devices used in this study (top panel) and corresponding images acquired (bottom panel) are shown.

Three handheld smartphone-enabled (oDocs visoScope, Remidio NMFOP, Volk iNview) and one handheld adaptor-detector based (Volk Pictor Plus) fundus camera/retinal imaging device were compared against a traditional table-top counterpart (Zeiss Visucam PRO^NM/FA^) (Fig. 1). Characteristics of each imaging modality are shown in Supplementary Table 2.

The Remidio Non-Mydriatic Fundus On Phone (NMFOP) (Bengaluru, India) is an infrared, smartphone-based fundus camera. Remidio NMFOP is a small system and can be used as a standalone handheld device or mounted on a slit lamp stand or used with its own table mounted stand, thus allowing the portability of the device [8]. Remidio NMFOP utilises specific technologies such as a patented annular illumination based optical design, infrared (IR) light and the voice coil motor of the smartphone camera to remove chromatic aberrations to improve image quality and capture [9]. The field of view (FOV) of the device is 40° [10]. At the time of this study, the cost of the device was approximately £4600 [6]. In this study we will refer to this imaging modality as the Remidio system.

Volk Pictor Plus (Mentor, Ohio, USA) is a non-mydriatic fundus camera with posterior (retinal) and anterior imaging modules. For the purpose of this study, only the retinal imaging module was utilised to visualise the fundus [11]. The device utilises a 40° FOV [12]. We also utilised the rubber cup that comes with the device to reduce the impact of extraneous light during image acquisition. At the time of this study, the cost of the device was approximately £4400 [12]. In this study we will refer to this imaging modality as the Pictor system.

Volk iNview (Mentor, Ohio, USA) fundus camera is an attachment to an Apple iPhone 6 s/6/5 s or iPod Touch (Gen 6). It uses a proprietary lens and an application that can be downloaded to take fundus images [13]. The device has a FOV of 50° [14]. At the time of this study, the cost of the device was approximately £700 (including the cost of the smartphone) [6]. In this study we will refer to this imaging modality as the iNview system.

The oDocs visoScope (Dunedin, New Zealand) is a 3D-printed adaptor which can be attached to a smartphone and together act as a retinal imaging device. It consists of an arm which places a lens at a specific distance away from the smartphone’s camera [15]. The FOV of the device is 45° [16]. At the time of this study, the cost of the device was approximately £260 (not including the cost of the smartphone) [17]. The smartphone camera used was a Google Pixel 3a. The best method for image acquisition was to capture a 4 K (3840 × 2160 pixels resolution) video at 30 frames per second. Highest quality fundus images were subsequently extracted from the videos for interpretation and analysis. In this study we will refer to this imaging modality as the oDocs system.

The Zeiss Visucam PRO^NM/FA^ (Oberkochen, Germany) is a table-mounted fundus camera and therefore was used as the “gold standard” for the purposes of this project, when comparing to the handheld fundus cameras. Visucam is a non-mydriatic camera which can capture images through pupils as small as 3.3 mm using its small pupil mode [18]. The device can utilise a FOV of 45° or 30° [19]. For this study, a 45° FOV was used. At the time of this study, the device cost approximately £14500 [20]. In this study we will refer to this imaging modality as the Zeiss system.

### Participant imaging

Stage 1 participants (*n* = 10) were recruited to assess all five fundus cameras/retinal imaging device for their success/failure rates of image acquisition in nonmydriatic and mydriatic settings. Image acquistion success was defined as successful capture of the ON head within the image frame. Participant experience with each device was also recorded. Mydriasis was achieved by instilling Tropicamide 1.0% eye drops in only the right eye of each participant, thus allowing capture and comparison of images in non-mydriatic and mydriatic settings. The imaging modalities utilised in this study were aimed at the posterior pole when acquiring images to include the optic disc and macula. Participants were asked to score the overall comfort of examination performed by each imaging modality on a 10-point Likert scale, considering flash intensity, proximity of the device and length of examination. The examiner also ranked ease of use of each instrument based on: (i) length of examination, (ii) portability and (iii) stability of acquisition.

Stage 2 participants with optic disc abnormalities (*n* = 8) and macular abnormalities (*n* = 10) were imaged by the top three scoring devices, for image acquisition success, as determined from stage one participants. Image capture was performed in only non-mydriatic settings.

### Clinician validation

A representative expert panel of clinicians was formed to validate and grade all captured images from both healthy participants (*n* = 10 panel members) and participants with ON abnormalities (*n* = 10 panel members) and macula abnormalities (*n* = 7 panel members). Clinicians had a mean experience in ophthalmology of 15 years. Five clinicians were of consultant grade, four were of senior specialist trainee grade (ST7) and one clinician was a post-certificate of completion of training (post-CCT) fellow. Clinicians validated images for their: (1) image quality, (2) gradeability of the ON, macula and vascular morphology (VM), (3) estimates for ON cup:disc (C:D) ratios and (4) ON or macula abnormality (binary choice: normal or abnormal). Image quality was assessed using a 10-point Likert scale.

Clinician estimates were then compared to true, measured ON C:D ratios to observe for their agreement and bias. Quantitative analysis was performed on all images captured in the study. This involved the measuring of the true C:D ratio using a custom script built in ImageJ software (version 1.48 (National Institutes of Health, Bethesda, Maryland, USA); available at http://rsbweb.nih.gov/ij/; accessed 15/06/2020).

### Statistical analysis

A D’Agostino-Pearson omnibus normality test was performed to assess the datasets for normal distribution using GraphPad Prism (version 7.04 for Windows, GraphPad Software, La Jolla California USA, www.graphpad.com). Sample size of 8 participants is required to achieve power of 80% to determine difference in quality ratings between imaging modalities (average SD = 1.95, α = 0.05, β = 0.2, mean difference = 2.9).

For parametric datasets, a one-way ANOVA test and a post-hoc Bonferroni’s multiple comparisons test, with a single pooled variance was performed using GraphPad Prism. For non-parametric datasets, a Kruskal–Wallis test and a post-hoc Dunn’s multiple comparisons test was performed using GraphPad Prism. For all analysis, a probability value of *p* ≤ 0.05 was considered statistically significant.

A Bland-Altman statistical test using GraphPad Prism was performed to observe for the agreement and bias between the true C:D ratios calculated by the ImageJ software and the clinician estimates. Diagnostic sensitivity and specificity for optic nerve head and macula abnormalities (binary classification: normal or abnormal) for the three devices (Remidio, Pictor and Zeiss) was also calculated together with the 95% confidence intervals.

## Results

### Success of image acquisition with and without mydriasis

**Fig. 2:**
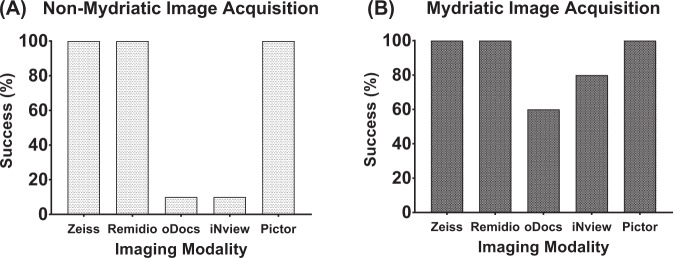
Imaging acquisition success. Bar charts demonstrating the percentage success rates for image acquisition from the devices utilised in the study in (**A**) non-mydriatic and (**B**) mydriatic settings.

Zeiss, Remidio and Pictor achieved a 100% success rate for image acquisition when utilised in both mydriatic and non-mydriatic settings. oDocs and iNview achieved low success rates (10%) when utilised in non-mydriatic settings, however, higher rates of success (60% and 80%, respectively), were achieved after mydriasis. This was comparatively lower than Zeiss, Remidio and Pictor in the mydriatic setting (Fig. 2). Examples of images acquired by each device is shown in Fig. 1.

### Image quality and gradeability

**Fig. 3:**
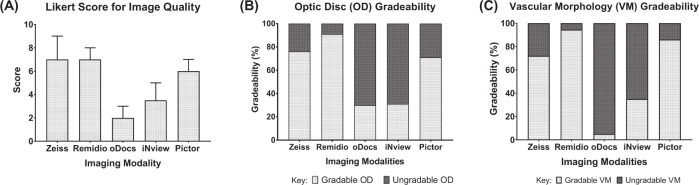
Clinician grading for imaging quality and gradeability. **A** Bar chart demonstrating the median Likert score for image quality for each imaging modality with error bars indicating the upper interquartile range for each corresponding median. Bar charts demonstrating the percentage gradeability of the (**B**) optic disc and (**C**) vascular morphology for each imaging modality.

Each device had different tones for the retina image output (Fig. 1). Zeiss produced images that were most “true” to colour for the retina, whilst image outputs from Remidio and Pictor demonstrated more “pink” and “red” tones, respectively (Fig. 1). Clinician derived quality scores were significantly different between the fundus imaging devices (Kruskal Wallis statistic = 272.6, *p* < 0.0001) (Fig. 3). Zeiss and Remidio had the highest image quality (median score = 7.0), followed by Pictor (median = 6.0), iNview (median = 3.5) and oDocs (median = 2.0) (Fig. 3). Compared to Zeiss, there was no difference in image quality for both Remidio and Pictor. However, iNview and oDocs had significantly lower quality scores in comparison to Zeiss (*p* < 0.0001) (Fig. 3).

Gradeability of the optic disc was highest for Remidio (91.1%) while lowest gradeability was seen with oDocs (30.0%) and iNview (31.0%). Similarly, gradeability of the VM in images was highest from Remidio (94.4%) and lowest in oDocs (5.1%) and iNview (35.0%). Zeiss and Pictor were considered having at least 70% of their images gradable for their optic disc and VM (Fig. 3).

### Participant experience

**Fig. 4:**
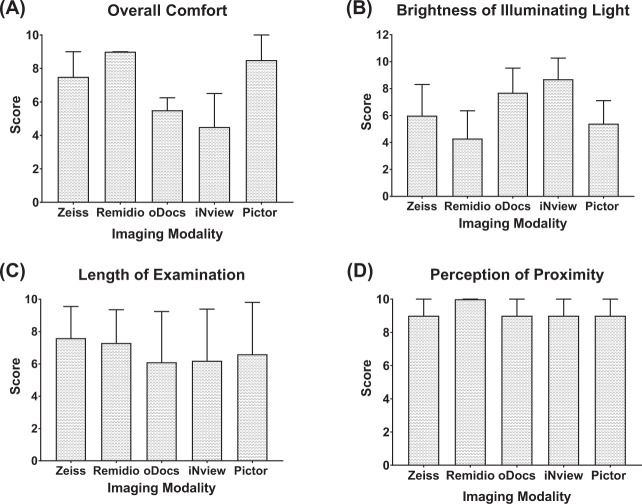
Comparsion of patient experience of each imaging modality. Bar charts demonstrating the median (**A**) overall comfort and (**D**) proximity scores of each imaging modality with error bars representing the upper interquartile range of the corresponding median. Bar chart displaying the mean (**B**) brightness and (**C**) length of examination scores of each imaging modality as given by participants with error bars corresponding to the upper standard deviation of each corresponding mean.

Overall comfort scores determined by the healthy participants were significantly different between the different instruments (Kruskal–Wallis statistic = 18.76, *p* = 0.0009) (Fig. 4). Compared to Zeiss, no significant difference in overall comfort scores were noted for all four handheld devices (*p* > 0.05). However, multiple comparisons revealed Remidio and Pictor to have significantly greater comfort than oDocs (*p* < 0.05). Remidio also had significantly greater overall comfort scores than iNview (*p* < 0.02). Zeiss, Remidio and Pictor all had significantly lower light intensities as reported by participants than iNview (*p* < 0.004). In addition, Remidio had significantly lower light intensity than oDocs (*p* < 0.0025) (Fig. 4).

### Examiner experience with image acquisition

All images were acquired by a single examiner to ensure consistency across imaging modalities. All devices except the oDocs had an upright image. The horizontally and vertically inverted image with oDocs provide an additional challenge during image acquisition. Examination time was shortest with Zeiss followed by Remidio, Pictor, iNview and oDocs, respectively. The examiner rated image acquisition stability highest with Zeiss followed by Remidio, Pictor, iNview and oDocs, respectively. Portability ranking by the examiner revealed highest portability with oDocs followed by iNview, Pictor, Remidio and Zeiss respectively. Examiner rankings are shown in Supplementary Table 3.

### Diagnostic sensitivity and specificity

Zeiss system had highest diagnostic sensitivity (Mean: 84.9%, 95% CI: 78.2–91.5%), followed by Pictor (Mean: 78.1%, 95% CI: 66.6–89.5%) and Remidio (Mean: 77.5%, 95% CI, 65.9–89.0%). We observe similar diagnostic specificity between the Zeiss system (Mean: 82.0%, 95% CI: 77.5–86.5%), Remidio (Mean: 79.0%, 95% CI: 73.7–84.3%) and Pictor (Mean: 83.0%, 95% CI: 79.5–86.5%).

### Agreement with reference standards for Cup:disc ratios

**Fig. 5:**
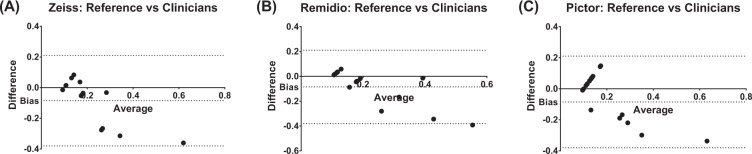
Comparison of clinician estimates of cup:disc ratio against reference standards. Figures demonstrating the results of the Bland–Altman test for agreement and bias performed for optic cup:disc ratios for (**A**) Zeiss, (**B**) Remidio NMFOP and (**C**) Pictor.

When comparing clinician estimates of C:D ratio against the reference standards determined computationally, the overall the best agreement and least bias was for Pictor (bias = −0.05 ± SD:0.16), followed by Remidio (bias = −0.07 ± 0.14) and Zeiss (bias = −0.09 ± SD:0.15). Remidio was the only modality that had outliers, with 2 of its 10 results not falling within the 95% confidence limits of agreement (Fig. 5).

## Discussion

In this study we report for the first time the comparative feasibility and utility of four handheld fundus cameras/retinal imaging device in relation to a traditional table-top fundus camera. We find that two (Remidio and Pictor) of the four handheld devices had equivalent image acquisition success and image quality in comparison to the table-top device (Zeiss). We explored patient preference and experience during image acquisition which revealed a similar result with Remidio and Pictor having similar or higher levels of acceptability in comparison to the table-top device. Finally, we identified good agreement between clinician estimates of C:D ratio for Remidio, Pictor and Zeiss and reference standards.

Zeiss, Remidio and Pictor all demonstrated 100% success rates for image capture in non-mydriatic settings. One possible explanation for the shared success was the availability of an IR or near-IR setting in each of these devices. IR light assisted retinal guidance systems have previously demonstrated success with retinal imaging in handheld fundus cameras [6]. Remidio utilises a custom-built circuit that enables the external flash to only have a delay of tens of microseconds to minimise pupil constriction [9]. In contrast, oDocs and iNview both demonstrated poor success rates in non-mydriatic settings. Both devices shared a common limitation, by utilising a constant, white light source whilst capturing fundus images, subsequently, constricting the pupils. Smartphone based applications to allow for adjustment of the light intensity and flash duration may aid in image capture in non-mydriatic settings [6].

The use of a constant light source was reflected in the participant scores which demonstrated that iNview was significantly brighter, and consequently less comfortable, than Zeiss, Remidio and Pictor. A recent publication by Zafar et al. (2018) highlighted the difficulties in imaging with modalities that use a bright light source, creating a challenge in examining participants with a degree of photophobia [21]. A further aid that may contribute to better image acquisition, specific to the Pictor imaging device, is the presence of a cup. This may reduce pupil constriction from external lights and act as a distance guide for the examiner. However, we did not find any difference in acquisition success or patient comfort between Pictor and Remidio.

For the clinician validation stage, highest scores for image quality were achieved by Zeiss, Remidio and Pictor. This corroborates previous literature which suggests that the image quality of Remidio is comparable to traditional table-top fundus cameras [22]. Pictor has also demonstrated high image quality in current literature [23].

Subtle differences such as the artificial tones to the images may influence clinician grading or favourability towards specific outputs. This has yet to be explored and requires further study. oDocs was associated with lowest image quality scores and this may be partly attributed to the light reflections on the lens. The image acquisition in this study was performed in a darkened room to: (1) minimise extraneous light, (2) reduce reflection on the lens and (3) improve natural pupil dilation. However, unlike other handheld devices, oDocs is not an enclosed system which may contribute to additional imaging artefacts and reduced quality.

The results of our study demonstrates that both Remidio and Pictor have similar, if not a higher, percentage of images that are considered gradable compared to a table-top camera. The high gradeability of images from Remidio and Pictor has allowed development of artificial intelligence-based screening methods for referrable diabetic retinopathy which relies of accurate gradeability of VM [24–26].

Agreement of clinician estimates of C:D ratio with ground truth data (derived computationally) for Remidio, Pictor and Zeiss have been shown in this study. Similarly, we describe similar diagnostic sensitivities between these three devices. This further re-enforces the ON head gradeability results for these devices and highlights the potential for use of the handheld devices (Remidio and Pictor) to not only screen for ON pathologies but also derive and monitor clinically relevant parameters such as C:D ratio. Previously, the use of Pictor in glaucoma C:D ratio determination has shown moderate inter-observer agreement [27]. The authors suggest that the potential contributory factors that reduced reproducibility may include poorer quality of images due to smaller pupils and high percentage of patients with significant cataracts. In our study none of our participants had any media opacity (based on slit lamp examination) and thus may partly explain the higher levels of agreement observed. Similarly, none of our participants had glaucoma thus overall variance of C:D ratio measures would be comparatively less. Interestingly, we observe lower bias associated with both hand-held devices (Pictor and Remidio) in comparison to the table-top device (Zeiss). This suggests that Remidio and Pictor are suitable to be utilised as an alternative to Zeiss in certain clinical scenarios.

The greater portability of these devices and their lower costs [6], imply that they are suitable for use on patients who have difficulties mobilising (bed-bound, home visits, rural settings) and in low-income countries. In addition, these devices operating successfully in non-mydriatic settings combined with their portability provide advantages in imaging paediatric population. Paediatric populations provide a challenge in imaging as they are more averse to pharmacological pupil dilation and long image capture times which are potentially overcome by the modalities discussed in this study [28].

## Conclusion

Our preliminary findings indicate the potential feasibility of handheld devices in comparison to table-top fundus imaging. Further studies with larger sample sizes and inclusion of different disorders are required for validation. Our results highlight that two of the handheld devices (Remidio and Pictor) provide equivalent imaging acquisition success (in both non-mydriatic and mydriatic setting) and quality comparable to a table-top fundus imaging device. We also show that quantitative parameters such as C:D ratio can be reliably derived from these handheld devices. The results from this study lay the groundwork for future studies exploring the use of handheld fundus cameras in various disorders and settings. Taken together, we anticipate the clinical utility of handheld devices could address the growing demand for imaging in different demographics (for example paediatrics), clinical environments (for example bed-bound patients) and development of tele-medicine capabilities.

### Summary

#### What was known before?

- Handheld cameras are available at significantly lower service costs in comparison to traditional table-top cameras.
- There are limited comparative studies looking at feasibility and clinical utility of handheld fundus cameras in relation to traditional table-top fundus cameras.

#### What this study adds?

- Two fundus cameras (Pictor and Remidio) demonstrated high success rates of image acquisition and high image quality and gradeability, comparable to results from table-top devices.
- Quantitative parameters such as C:D ratio can be reliably derived from some handheld devices.
- Clinical utility of handheld devices could address the growing demand for imaging in different demographics (e.g. paediatric), clinical environments (e.g. bed-bound patients) and development of tele-medicine applications.

## Supplementary information

Supplementary table 1

Supplementary table 2

Supplementary table 3
