# Correction: Feasibility and clinical utility of handheld fundus cameras for retinal imaging

**DOI:** 10.1038/s41433-022-02041-2

**Published:** 2022-05-25

**Authors:** Susmit Das, Helen J. Kuht, Ian De Silva, Sundeep S. Deol, Lina Osman, Joyce Burns, Nagini Sarvananthan, Usman Sarodia, Bharat Kapoor, Tahir Islam, Raghavan Sampath, Alicia Poyser, Vasileios Konidaris, Rossella Anzidei, Frank A. Proudlock, Mervyn G. Thomas

**Affiliations:** 1 Leicester Medical School, College of Life Sciences, University of Leicester, George Davis Centre, 15 Lancaster Rd, Leicester, LE1 7HA UK; 2 The University of Leicester Ulverscroft Eye Unit, Department of Neuroscience, Psychology and Behaviour, University of Leicester, Leicester, LE2 7LX UK; 3 Department of Ophthalmology, University Hospitals of Leicester, Leicester Royal Infirmary, Leicester, LE1 5WW UK

**Keywords:** Medical imaging, Medical research

Correction to: *Eye* 10.1038/s41433-021-01926-y, published online 12 January 2022

List of changes:

1. Abstract:

a. Background/objectives

Original: We compare the feasibility and clinical utility of four handheld fundus cameras (Remidio NMFOP, Volk Pictor Plus, Volk iNview, oDocs visoScope) to a table-top camera (Zeiss VisucamNM/FA).

Change to: We compare the feasibility and clinical utility of four handheld fundus cameras/retinal imaging devices (Remidio NMFOP, Volk Pictor Plus, Volk iNview, oDocs visoScope) to a table-top camera (Zeiss VisucamNM/FA).

b. Methods

Original: Healthy participants (*n* = 10, mean age ± SD = 21.0 ± 0.9 years) underwent fundus photography with five fundus cameras to assess success/failure rates of image acquisition.

Change to: Healthy participants (*n* = 10, mean age ± SD = 21.0 ± 0.9 years) underwent fundus photography with five devices to assess success/failure rates of image acquisition.

2. Introduction:

a. Original: In our study, we compare four handheld fundus cameras to a table-top counterpart. We assess the cameras for their feasibilities of image acquisition, image quality and gradeability, and participant experience.

Change to: In our study, we compare four handheld fundus cameras/retinal imaging device to a table-top counterpart. We assess the devices for their feasibilities of image acquisition, image quality and gradeability, and participant experience.

3. Materials and methods:

a. Imaging modalities:

Original: Three handheld smartphone-enabled (oDocs visoScope, Remidio NMFOP, Volk iNview) and one handheld adaptor-detector based (Volk Pictor Plus) fundus camera were compared against a traditional table-top counterpart.

Change to: Three handheld smartphone-enabled (oDocs visoScope, Remidio NMFOP, Volk iNview) and one handheld adaptor-detector based (Volk Pictor Plus) fundus camera/retinal imaging device were compared against a traditional table-top counterpart.

Original: The oDocs visoScope (Dunedin, New Zealand) is a 3D-printed fundus camera that can be attached to a smartphone.

Change to: The oDocs visoScope (Dunedin, New Zealand) is a 3D-printed adaptor which can be attached to a smartphone and together act as a retinal imaging device.

b. Participant imaging

Original: Stage 1 participants (*n* = 10) were recruited to assess all five fundus cameras for their success/failure rates of image acquisition in nonmydriatic and mydriatic settings.

Change to: Stage 1 participants (*n* = 10) were recruited to assess all five fundus cameras/retinal imaging device for their success/failure rates of image acquisition in nonmydriatic and mydriatic settings.

c. Figure 1 legend

Original: Overview of table-mounted and handheld fundus cameras and fundus images. The fundus cameras used in this study (top panel) and corresponding images acquired (bottom panel) are shown.

Change to: Overview of table-mounted and handheld fundus cameras/retinal imaging devices and fundus images. The devices used in this study (top panel) and corresponding images acquired (bottom panel) are shown.

4. Discussion

a. Original: In this study we report for the first time the comparative feasibility and utility of four handheld fundus cameras in relation to a traditional table-top fundus camera.

Change to: In this study we report for the first time the comparative feasibility and utility of four handheld fundus cameras/retinal imaging device in relation to a traditional table-top fundus camera.

The original article has been corrected.

